# Effects of albendazole combined with TSII-A (a Chinese herb compound) on optic neuritis caused by *Angiostrongylus cantonensis* in BALB/c mice

**DOI:** 10.1186/s13071-015-1214-6

**Published:** 2015-11-25

**Authors:** Feng Feng, Ying Feng, Zhen Liu, Wei-Hua Li, Wen-Cong Wang, Zhong-Dao Wu, Zhiyue Lv

**Affiliations:** Parasitology Department of Zhongshan School of Medicine, Sun Yat-sen University, Guangzhou, 510080 China; Key Laboratory of Tropical Disease Control (SYSU), Ministry of Education, Guangzhou, 510080 China; Histology and Embryology Department of Zhongshan School of Medicine, Sun Yat-sen University, Guangzhou, 510080 China; Zhongshan Ophthalmic Center, SunYat-sen University, Guangzhou, 510080 China

**Keywords:** Optic neuritis, *Angiostrongylus cantonensis*, Albendazole combined with TSII-A, BALB/c mice

## Abstract

**Background:**

*Angiostrongylus cantonensis* (*A. cantonensis*) infection can lead to optic neuritis, retinal inflammation, damage to ganglion cells, demyelination of optic nerve and visual impairment. Combined therapy of albendazole and dexamethasone is a common treatment for the disease in the clinic, but it plays no role in vision recovery. Therefore, it has been necessary to explore alternative therapies to treat this disease. Previous studies reported the neuro-productive effects of two constituents of Danshen (a Chinese herb)-tanshinone II-A (TSII-A) and cryptotanshinone (CPT), and this study aims to evaluate the impacts of TSII-A or CPT combined with albendazole on optic neuritis caused by *A. cantonensis* infection in a murine model.

**Methods:**

To assess the effects of TSII-A or CPT combined with albendazole on optic neuritis due to the infection, mice were divided into six groups, including the normal control group, infection group and four treatment groups (albendazole group, albendazole combined with dexamethasone group, albendazole combined with CPT group and albendazole combined with TSII-A group). The infection group and treatment groups were infected with *A. cantonensis*and the treatment groups received interventions from 14 dpi (days post infection), respectively. At 21 dpi, the visual acuity of mice in each group was examined by visual evoked potential (VEP). The pathologic alteration of the retina and optic nerve were observed by hematoxylin and eosin (H&E) staining and transmission electronic microscopy (TEM).

**Results:**

Infection of *A. cantonensis* caused prolonged VEP latency, obvious inflammatory cell infiltration in the retina, damaged retinal ganglions and retinal swelling, followed by optic nerve fibre demyelination and a decreasing number of axons at 21 dpi. In treatment groups, albendazole could not alleviate the above symptoms; albendazole combined with dexamethasone lessened the inflammation of the retina, but was futile for the other changes; however, albendazole combined with CPT and albendazole combined with TSII-A showed obvious effects on the recovery of prolonged VEP latency, destruction and reduction of ganglion cells, optic nerve demyelination and axon loss. Compared with albendazole-CPT compound, albendazole combined with TSII-A was more effective.

**Conclusions:**

The current study demonstrates that albendazole combined with TSII-A plays a more effective role in treating optic neuritis caused by *A. cantonensis* in mice than with dexamethasone, as applied in conventional treatment, indicating that albendazole combined with TSII-A might be an alternate therapy for this parasitic disease in the clinic.

## Background

Angiostrongyliasis is a food-borne parasitic disease caused by *Angiostrongylus cantonensis*. The third-stage larva of *A. cantonensis*mainly invades the central nerve system and causes meningitis, spinal meningitis, encephalitis, myelitis and optic neuritis [1–4]. There are ongoing reports of optic neuritis cases caused by *A. cantonensis*infection*.* In a previous review, we made an extensive literature survey on ocular angiostrongyliasis with 42 sporadic cases [5]. Moreover, Punyagupta et al. [6] analysed the clinical features of 484 cases of typical eosinophilic meningitis and found that 16 % of patients had visual impairment, while 12 % had an optic disc abnormality such as papilledema and atrophy, which implied that the actual incidence of ocular angiostrongyliasis is likely much higher than commonly appreciated (1 %) [6].

Oral steroids such as dexamethasone combined with an anti-helminthic agent like albendazole have been the most effective and common therapy clinically so far for the damage to vision caused by this parasite [7], for the significant improvement of intraocular inflammation and visual acuity. However, this treatment does not always achieve complete recovery of vision, which severely interferes with the quality of life of patients [8] and hence leaves an unsolved problem for researchers nowadays.

Our previous study tried to explore the pathologic changes and effective treatments for optic neuritis caused by *A. cantonensis* infection in animal models. We considered BALB/c mice as an ideal model for *A. cantonensi*s-induced optic neuritis for the following representative pathological changes: inflammatory cell infiltration in retina and optic nerve adventitia followed by distinct optic nerve fibre demyelination and retinal ganglion swelling. Moreover, tribendimidine (TBD) therapy can significantly ease retina and optic nerve inflammation, but vision did not recover owing to the ineffective rescue of the damaged ganglion cells and myelin sheath [9]. Besides, we also reported thatalbendazole-dexamethasone compound, as the conventional therapy, has little effect on the pathologic damage to optic nerve caused by *A. cantonensis* in infected rats [10].

All the unsolved problems mentioned above have urged us to prioritize research into a more effective treatment for *A. cantonensis*-induced optic neuritis. In this study, two constituents of Danshen (Salviamiltiorrhiza), tanshinone II-A (TSII-A) and cryptotanshinone (CPT), were combined with albendazole separately to treat the disease. Danshen is a type of traditional Chinese herb, which has drawn extensive attention as an effective therapeutic against a number of diseases including atherosclerosis, hyperlipidemia, hypertension, stable angina pectoris, myocardial infarction, coronary artery disease, diabetes and metabolic syndrome, supported by published papers, approved patents and clinical trials in the United States. TSII-A and CPT are the most abundant and well-studied constituents of Danshen, which exerts antioxidant and anti-inflammatory effects on many experimental animal models, achieving potential protective effects against atherosclerosis, cardiac hypertrophy, cardiac fibrosis, diabetes, neurodegenerative diseases, and various kinds of cancers [11, 12]. Attributing to their neuro-protective effect [13–15], they were applied in our study to protect the damage of ganglion cells caused by *A. cantonensis* in infected mice so as to rescue the loss of vision. Our results showed TSII-A or CPT combined with albendazole was more effective than the other two treatment groups, including the traditional albendazole-dexamethasone group. They could reverse the damage and loss of ganglion cells, reduce the demyelination of optic nerve, and return the number and diameter of nerve fibres to normal, and hence significantly improved vision acuity. Between them, TSII-A was more effective than CPT, indicating that albendazole combined with TSII-A can be a more effective therapeutic treatment than the albendazole-dexamethasone compound to treat optic neuritis caused by *A. cantonensis* and should be applied in clinic. Our study provides an alternative medication therapy for optic neuritis caused by *A.cantonensis*.

## Methods

### Infection of mice with larvae of *A. cantonensis*

BALB/c mice (20–40 g body weight) were purchased from the animal centre laboratory at Sun Yat-sen University (Guangzhou, China). The Institutional Animal Care and Use Committee approved all animal procedures. The third stage larvae (L3) of *A. cantonensis* were collected and used to infect the mice as described in the methods section of our previously published paper [8]. The animals were divided into six groups: normal control group, infected group, and four treatment groups (albendazole group, albendazole combined with dexamethasone group, albendazole combined with CPT group and albendazole combined with TSII-A group). There were six animals in every group. Except for the normal control group, the other five groups were infected with 30 L3, respectively. After 14 dpi (days post infection), the treatment groups were fed with albendazole (20 mg/kg/d), albendazole combined with dexamethasone (0.5 mg/kg/d), albendazole combined with CPT (50 mg/kg/d) and albendazole combined with TSII-A (50 mg/kg/d), respectively. The normal and infected groups were treated with the same amount of DMSO as control. All the medicines or DMSO were administered via gastric gavage to mice once per day for 7 days.

### Electrophysiological recordings

In consideration of the following VEP recording, all mice in the six groups were anesthetized and secured to a moveable platform at 21 dpi. In VEP recording, stainless steel screws were implanted 3 mm lateral to the lambda and 5 mm behind the bregma. Reference electrodes were placed 2 mm lateral to the lambda and 2 mm in front of the bregma. Transient VEPs were evoked by single-flash stimulation (1.3 Hz, 12 ms). VEP signals were recorded by a commercial system (Roland Consult GmbH, Brandenburg, Germany).

### Histopathological observation

Following sacrifice of the mice in the six groups at 21 dpi by cervical dislocation, paraffin embedded sections were prepared from isolated optic nerves and eyeballs. Optic neuritis was detected by the presence of inflammatory cell infiltration on hematoxylin and eosin (H&E) staining and observed by microscopy (Olympus, Japan). Retina thickness was measured using Image Pro Plus 6.0 (Media Cybernetics, USA). Demyelination detection was carried out using a 300 KV transmission electronic microscope (FEI, USA) and the number and diameter of axons were measured using Image Pro Plus 6.0.

### Immunohistochemical analysis

Mice were anaesthetized and perfused with 4 % paraformaldehyde in PBS. Eyeballs of mice in every group were fixed with 4 % paraformaldehyde overnight, equilibrated in 15 % and then 30 % sucrose solutions in PBS. Later, eyeballswere cut into15 μm thick sections at −20 °C and mounted on glass slides. Sections were then blocked with 3 % bovine serum albumin (BSA) at room temperature for 1 h before incubation with rabbit anti-mouse Brn-3A (1:125 dilution, Millipore, USA) monoclonal antibody in 1 % BSA at 37 °C for 1 h. Sections were washed three times in PBS, incubated with fluorescein isothiocyanate (TRITC)-labeled secondary antibody (Abcam, Cambridge, UK), diluted 1:500 in 1 % BSA at 37 °C for 1 h, and washed again in PBS. Then nuclei were stained with DAPI (1:1,000 diluted, Beyotime Biotechnology, Haimen, China) for 5 min. Specimens stained without the primary antibody were used as negative controls. The sections were then observed under a confocal microscope.

### Statistics

One-way ANOVA was used to compare VEP P2 latency time, retinal thickness, axon numbers, diameter of optic nerve fibres and ganglion cellcount among different groups, and a *p*-value of less than 0.05 was considered to be statistically significant. Statistics were performed using IBM SPSS statistics 19 (SPSS Inc, USA).

## Results

### Albendazole combined with TSII-A (a Chinese herb compound) has a curative effect on optic neuritis-induced vision impairment caused by *A. cantonensis*

**Fig. 1:**
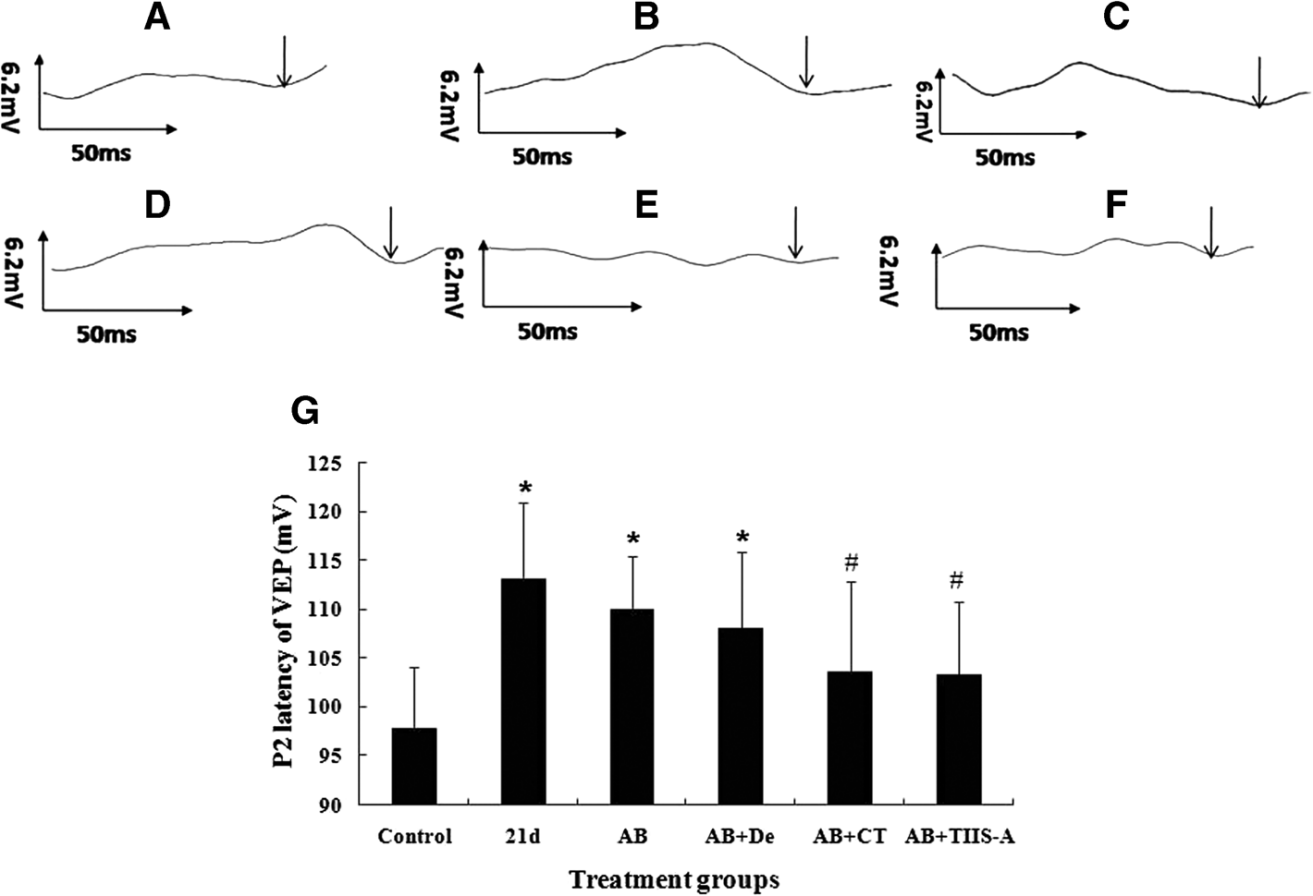
Data are mean ± SEM for P2 latency alterations of VEP in in different groups. **a**–**f**: P2 latency of VEP. Black arrow indicates P2 latency (ms).** a**: Normal group. **b**: Infected group by *A. cantonensis* (21 dpi). P2 latency is prolonged (>100 ms, ↓points). **c**: Albendazole (AB) treatment for 7 days after 14 dpi with *A. cantonensis*. P2 latency still is prolonged (>100 ms, ↓points). **d**: Albendazole combined with Dexamethasone (De) treatment for 7 days after 14dpi with *A. cantonensis*. P2 latency is still prolonged (>100 ms, ↓points). **e**: Albendazole combined with CPT treatment for 7 days after 14 dpi with *A. cantonensis*. P2 latency recovered to normal. **f**: Albendazole combined with TSII-A treatment for 7 days after 14 dpi with *A. cantonensis*. P2 latency recovered to normal. **g**: Data are mean ± SEM of P2 latency of mice in different treatment groups for 7 days after 14 dpi with *A. cantonensis*. *Statistically significant when compared with normal control (*P* < 0.05); #Statistically significant when compared with 21 dpi with *A. cantonensis*, *n* = 5 per group, *P* < 0.05

VEP measurements were performed to diagnose optic neuritis *in vivo*. In the present study, P2 latency of VEP of mice in the infection group became elongated at 21dpi (Fig. 1b, d) compared to normal control group (Fig. 1a). After treatment with albendazole and conventional albendazole-dexamethasoneprocedure, VEP of infected mice did not significantly decrease (Fig. 1c, d). Albendazole combined with CPT decreased the latency of VEP slightly, but did not regain the normal level (Fig. 1d, e). Nevertheless, albendazole combined with TSII-A was able to reverse the damaged vision and rehabilitate elongated P2 latency of VEP to basement line value (Fig. 1d, f).

### Albendazole combined with TSII-Acan alleviate the damage to ganglion cells

**Fig. 2:**
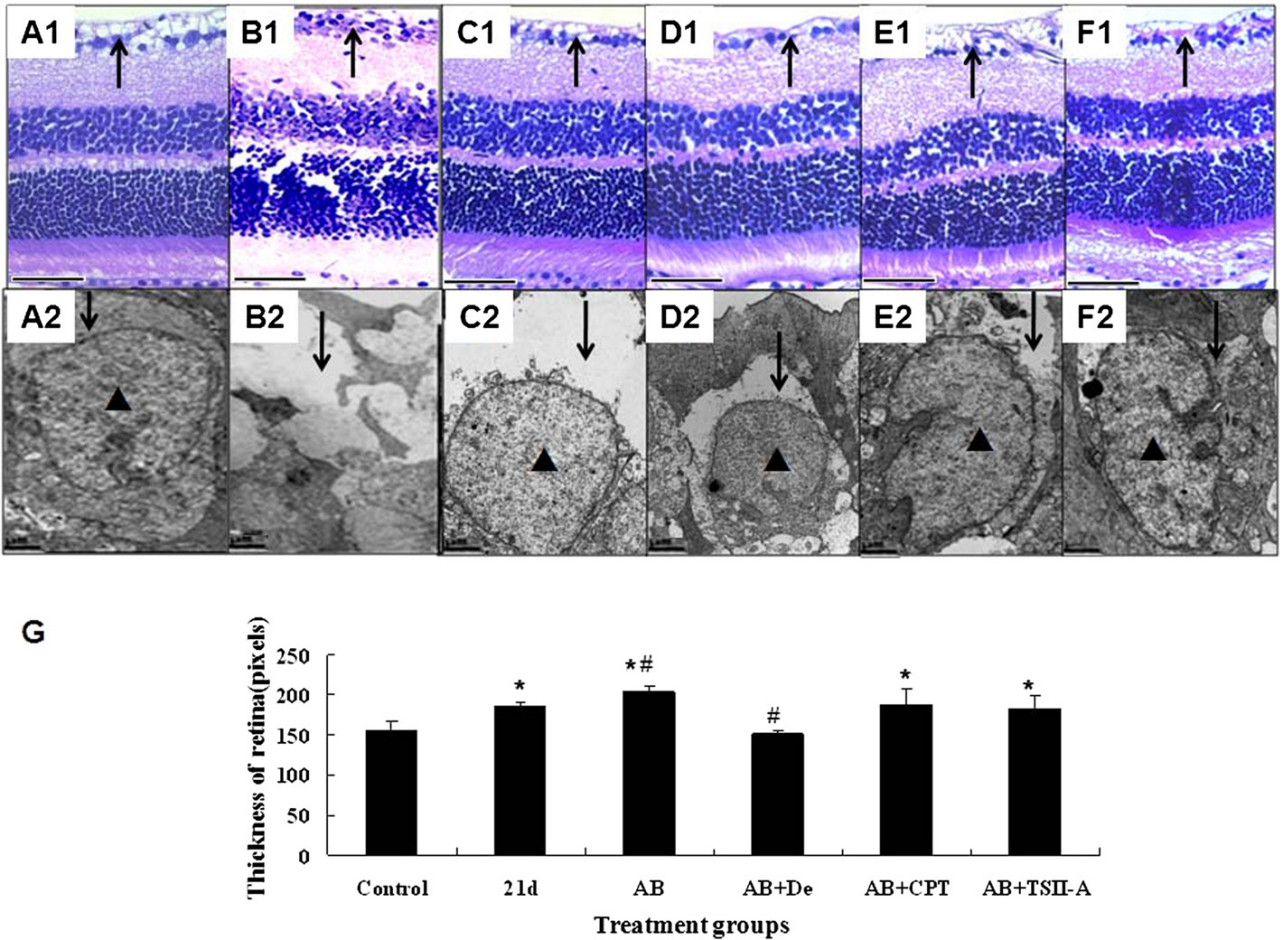
The alteration of mouse retina and ganglion cells before and after treatment regimen following infection with *A. cantonensis*. **a1-f1**: retina in different treatment groups with HE staning. bar = 100 μm.↑ points ganglion cell layer of retina. **a2-f2**: Electron microscopy images of ganglion cells in different treatment groups. ↓ points cytoplasm of ganglion cells; ▲ points the nucleusof ganglion cells. bar = 1 μm. **a1-a2**: retina and ganglion cell in normal group. There was a layer of ganglion cells (↑ in A1). The cytoplasm and nucleus of ganglion cells were intact (↓ and ▲ in A2). **b1-b2**: retina and ganglion cell in groups infected with *A. cantonensis* at 21 dpi. The ganglion cell layer showed inflammatory cell infiltration (↑ in B1). Swelling was pronounced in cytoplasm of ganglion cells (↓in B2) and nucleus diminished. **c1-c2**: Albendazole (AB) treatment for 7 days after 14 dpi with *A. cantonensis*. Inflammation was alleviated in ganglion cell layer of retina (↑ in C1). Swelling can still be observed in cytoplasm of ganglion cell (↓ in C2) with an intact nuclear as usual (▲ in C2). **d1-d2**: Albendazole combined with Dexamethasone treatment (AB + De) for 7 days after 14 dpi with *A. cantonensis*. The inflammation of the retina almost resolved (↑ in D2). Swelling of ganglion cytoplasm relieved (↓ in C2). The nucleus was still obviously observed (▲ in D2). **e1-e2**: Albendazole combined with CPT (AB + CPT)treatment for 7 days after 14 dpi with *A. cantonensis*. Inflammation was alleviated in ganglion cell layer of retina (↑ in E1). Swelling decreased greatly in cytoplasm of ganglion cell (↓ in E2). **f1-f2**: Albendazole combined with TSII-A (AB + TSII-A) treatment for 7 days after 14 dpi with *A. cantonensis*. Inflammation was alleviated slightly in ganglion cell layer of retina (↑ in F1). Swelling diminished in cytoplasm of ganglion cell (↓ in F2) and the cells regained normal structure as in A2. **g**: Retinal thickness measured under a light microscope in normal group, 21 dpi infection group, albendazole treatment group (AB), albendazole combined with dexamethasone (AB + De) treatment group, albendazole combined with CPT (AB + CPT) treatment and albendazole combined with TSII-A (AB + TSII-A) treatment group. Data are mean ± SEM. *Statistically significant when compared with control (0 dpi) (*P* < 0.05); # Statistically significant when compared with 21 dpi (*P* < 0.05)

Histopathological examination showed inflammatory cell infiltration in the ganglion cell layer (Fig. 2b1) and swollen retina (Fig. 2b2) at 21 dpi. Albendazole applied alone failed to diminish the inflammation but contrarily aggravated the swelling of the retina (Fig. 2c1, g). It failed to prevent the damage to the structure of ganglion cells, (Fig. 2c2). Albendazole combined with dexamethasone alleviated inflammation and swelling of the retina significantly (Fig. 2d1, g), but did not show obvious curative effects on the damaged ganglion cells (Fig. 2d2). Albendazole combined with CPT showed a slight anti-inflammatory effect on the retina (Fig. 2e1) and can alleviate the damage to ganglion cells; however, the cytoplasm swelling still existed (Fig. 2e2). On examining inflammatory changes, albendazole combined with TSII-A group could not reduce inflammationof the retina (Fig. 2f1), but could recover the damaged ganglion cell to normal structure efficaciously (Fig. 2f2).

### Albendazole combined with TSII-A can rescue the loss of ganglion cells

**Fig. 3:**
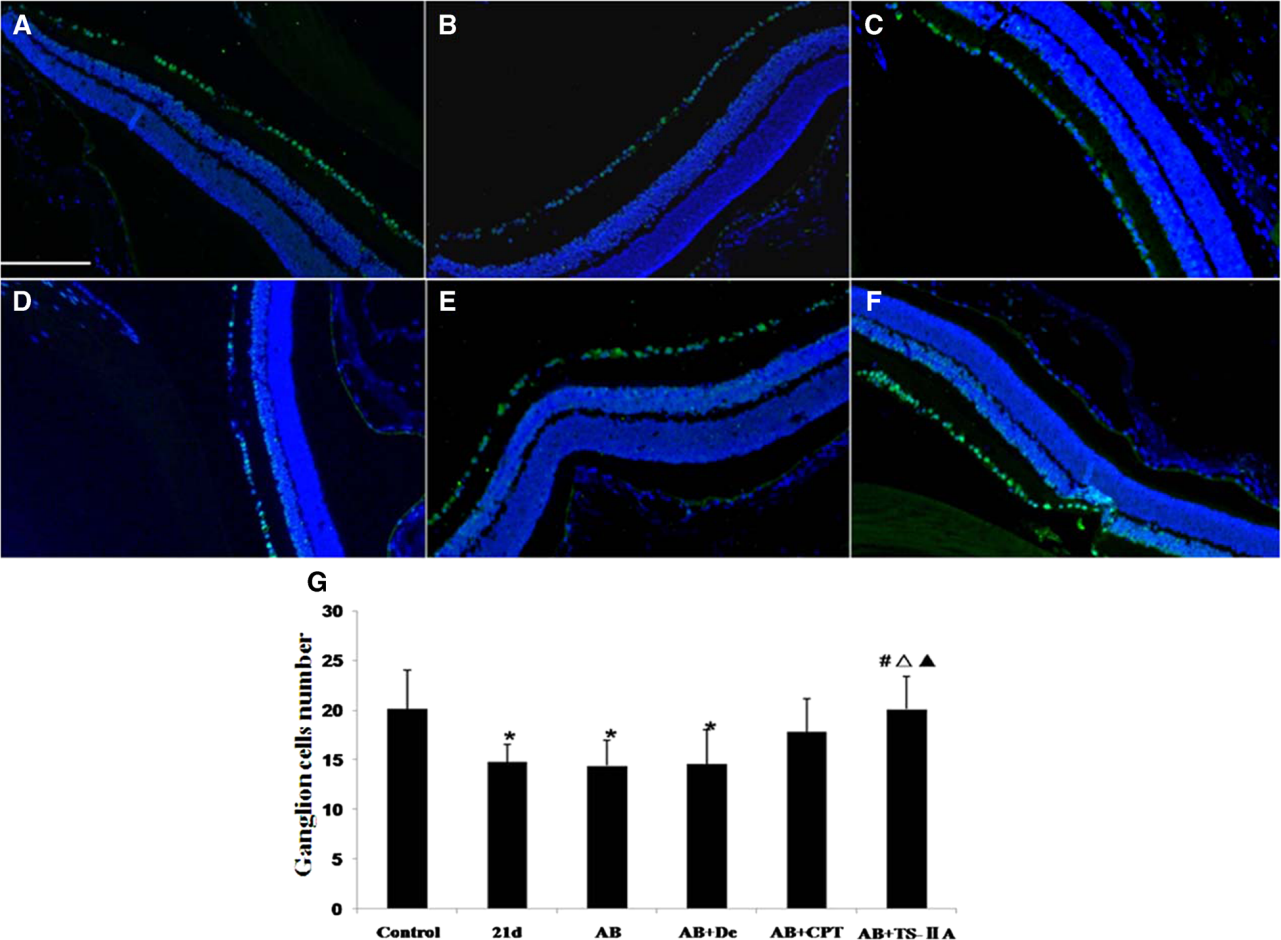
TSII-A promotes RGC survival of mice infected with *A. cantonensis*. **a-f**: Immunofluorescence reinforced the image of RGC cells labeled with Brn-3A (Green). Scale bar = 100 μm. **a** Normal control. **b**: Untreated mice infected with *A. cantonensis* (21 dpi). **c**: Albendazole (AB) for 7 days after 14 dpi with *A. cantonensis*. **d**: Albendazole combined with Dexamethasone (AB + De) for 7 days after 14 dpi with *A. cantonensis*. **e**: Albendazole combined with CPT (AB + CPT) for 7 days after 14 dpi with *A. cantonensis*. **f**: Albendazole combined with TSII-A (AB + TSII-A) for 7 days after 14 dpi with *A. cantonensis*. **g**: Data are mean ± SEM of ganglion cell numbers in normal group, untreated group for 21 dpi with *A. cantonensis*, and treated group for 7 days after 14 dpi with *A. cantonensis* infection, respectively. *Statistically significant when compared with control (0 dpi); # statistically significant when compared with 21 dpi; △ statistically significant when compared with albendazole treatment group, ▲ statistically significant when compared with Albendazole combined with Dexamethasone treatment group; *N* = 3 coverslips per group, with 5fields analyzed per coverslip, *P* < 0.05

The former experiment examined the pathological changes of retina and ganglion cells before and after treatment, indicating that TSII-A could achieve the best protective effect towards the ganglion cell damage caused by *A. cantonensis* compared with other groups. Next, we made further efforts to evaluate the protective effect of different therapies on ganglion cells by immunofluorescence-oriented cytometry. Our results demonstrated that *A. cantonensis* infection led to noteworthy ganglion cell loss (Fig. 3b, g). Albendazole or albendazole-dexamethasone treatment could not rescue the loss of ganglion cells (Fig. 3c, d, g) whereas albendazole combined with CPT (Fig. 3e, g) and albendazole combined with TSII-A can both effectively prevent the death of ganglion cells caused by *A. cantonensis*, and the curative effect of the former was more apparent (Fig. 3f, g).

### Albendazole combined with TSII-A can reverse the damage to nerve fibres

**Fig. 4:**
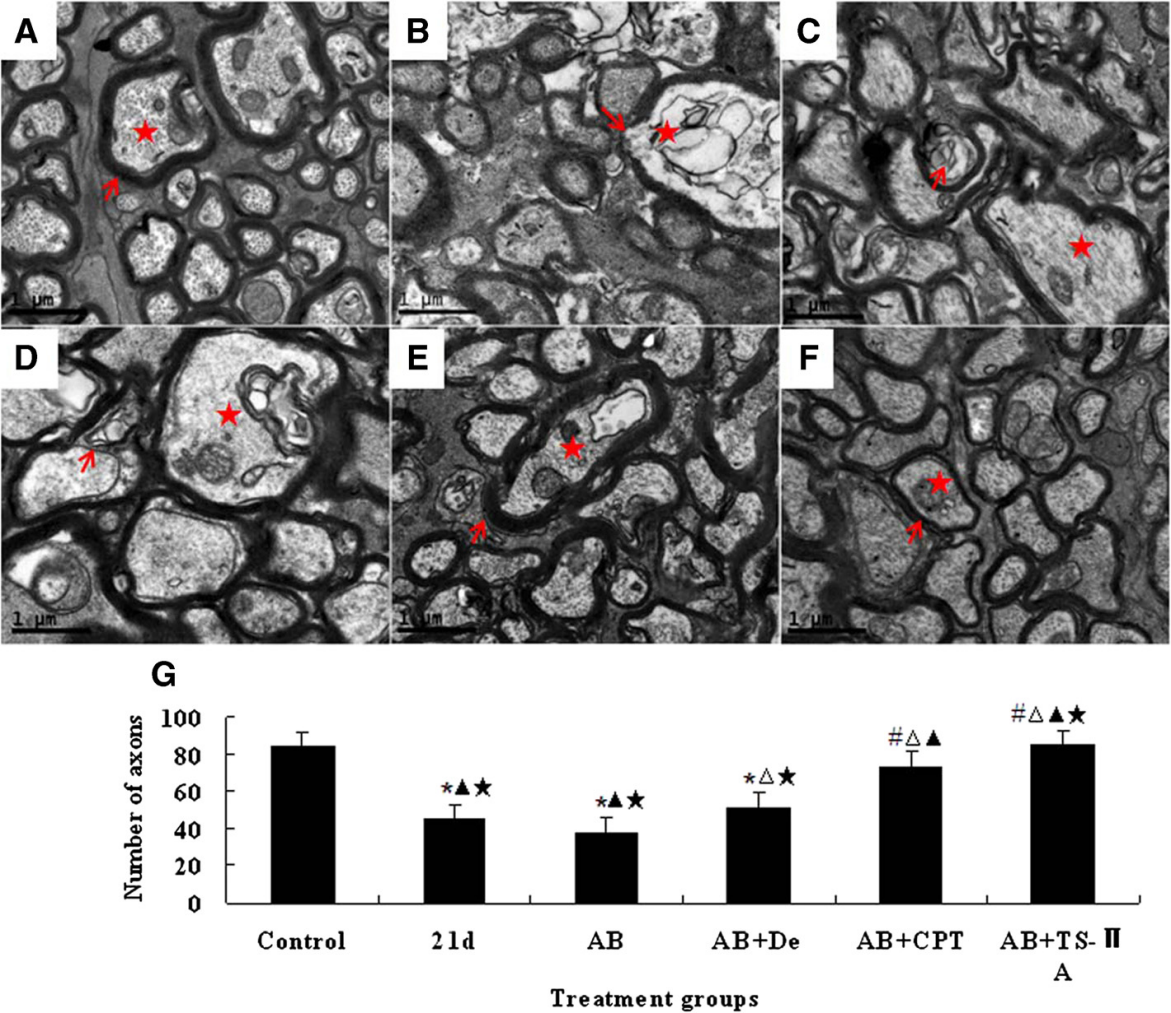
Histological alteration of optic nerve in *A. cantonensis*-infected mice after different treatments. **a-f**: Electron microscopy images of optic nerve, scale bar = 1 μm. ↑ points to myelin sheath; ★ marks axon. **a**: normal group. The myelin sheathes and axons are intact. **b**: infected group by *A. cantonensis* (21 dpi). Demyelination (red ↑) and impaired axons (red ★) can be observed. **c**: Albendazole (AB) treatment for 7 days after 14 dpi with *A. cantonensis*. Demyelination become lessened, but still can be observed. **d**: Albendazole combined with Dexamethasone (AB + De) treatment for 7 days after 14 dpi with *A. cantonensis*. Demyelination become lessened, but still can be observed (red ↑ and ★). **e**: Albendazole combined with CPT treatment for 7 days after 14 dpi with *A. cantonensis*. The myelin sheathes regain intact structure (red ★), but swelling can be observed in axons (red ). **f**: Albendazole combined with TSII-A treatment for 7 days after 14 dpi with *A. cantonensis*. Demyelinated axons almost recover to intact structure (red ↑ and ★). Scale bar = 1 μm. **g**: Data are mean ± SEM of axon numbers in different treatment groups for 7 days after 14 dpi with *A. cantonensis*, respectively. *Statistically significant when compared with control (0 dpi); # statistically significant when compared with 21 dpi; △ statistically significant when compared with albendazole treatment group, ▲ statistically significant when compared with albendazole combined with Dexamethasone treatment group; ★ statistically significant when compared with albendazole combined with CPT treatment group. *n* = 3 per group, with 5 fields analysed per section. *P* < 0.05

The results of TEM showed that noteworthy demyelination appeared in the optic nerve (Fig. 4b), accompanied by a reduction in the number of axons and an increased diameter of myelin sheath (Fig. 4d, g) at 21 dpi. Although albendazole can alleviate the demyelination, it cannot help to regain the number of axons (Fig. 4d, g). Results from the albendazole combined with dexamethasone treatment group can also mitigate demyelination and axon destruction, and can increase the number of axons to some extent but not to the normal level (Fig. 4d, g). However, in the albendazole-CPT or albendazole-TSII-A treated group, both the structure of the myelin sheath and axon and the number of axons regained normal levels (Fig. 4e, f), and the latter was more effective (Fig. 4g). These results indicated that albendazole combined with TSII-A could not only alleviate the demyelination of the optic nerve, but also contributed to the remyelination of axons by increasing their number.

## Discussion

In this study, we compared the compound of albendazole and the constituents of Danshen (one Chinese herb), TSII-Aand CPT, in the treatment of optic neuritis caused by *A. cantonensis*. Previous studies showed that Danshen derivatives, TSII-A and CPT, exert antioxidant and anti-inflammatory actions in many experimental animal models. In addition, neuroprotective effects of TSIIA have been reported in rats, mice and *in vitro* assays. As a result, we chose TSII-A combined with albendazole to treat the disease and expected to attain a better therapeutic effect compared with the conventional albendazole - dexamethasone combination therapy. Our results showed that albendazole combined with TSII-A may be preferable to other therapeutic methods in pathologic change improvement and visual acuity recovery. The evidence was as follows.

Firstly, the detection of VEP demonstrated that TSII-A contributed to the vision recovery in optic neuritis caused by *A. cantonensis*. VEP is the most sensitive method to examine optic neuropathy and is applied as an important index to diagnose optic neuritis and evaluate the functional status from retina ganglion cells to optic cortex [16]. Moreover, it may be used as a real measurement of demyelination in the rat model of optic neuritis [17]. In the present study, VEP latency of mice at 21 dpi appeared obviously delayed, which may have intimate correlation with the damage of optic nerve fibres and ganglion cells. While there was no obvious VEP recovery with sole albendazole treatment and albendazole combined with dexamethasone treatment, VEP latency returned to normal in CPT and TSII-A combined with albendazole treatment groups. The results also indicate that TSII-A can rescue the function of ganglion cells and parts of optic nerve fibres, which have been damaged by the infection with *A. cantonensis.*

Secondly, the results of immunofluorescence also proved the curative effect of albendazole combined with TSII-A treatment in optic neuritis caused by *A. cantonensis.* At 21 dpi, the number of ganglion cells seen in the infection group decreased sharply compared to the normal control group. Ganglion cells are the most important neurons in the retina and their axons form the optic nerve. The damage of ganglion cells will cause serious vision impairment [18]. Sole albendazole treatment and albendazole combined with dexamethasone treatment increased the ganglion cell number slightly, whereas CPT or TSII-A combined with albendazole treatment were shown to help almost regain the initial number of ganglion cells, and the latter was more effective, indicating that albendazole combined with TSII-A can rescue the loss of ganglion cells caused by *A. cantonensis* infection significantly. Previous studies showed TSII-A played a vital role in protecting neurons such as dopaminergic neurons and hippocampal neurons [19, 20], and may inhibit thebeta-amyloid-induced toxicity in rat cortical neurons [21, 22]. In addition, TSII-A also has a protective effect on the cranial nerves when administered during the initial stages of cerebral ischemia [14]. Our results indicated TSII-A can improve ganglion cell impairment caused by *A. cantonensis,* however, the mechanisms involved need to be further explored*.*

Finally, the present study has clearly shown that albendazole combined with TSII-A group rescued the damaged ganglion cells and optic nerves. The infection group showed severe retinal inflammation, ganglion cell destruction and optic nerve demyelination, followed by decreasing axon number and swelling of nerve fibres, which indicate that *A. cantonensis* can definitely cause optic neuritis and structural alteration of retina and optic nerve [9]. Sole albendazole treatment cannot improve the inflammation or damaged ganglion cells, but on the contrary aggravated the inflammation of eyes due to thickened retina. The reason may be that the decomposition debris of dead worms killed by albendazole leads to an immune reaction of hosts [23], and enhances the inflammation of the brain, which spreads into the eyes through the optic nerve. Moreover, it was either ineffective to lessen the damage to the optic nerve because it did not increase the number of axons in the optic nerve compared with the untreated group. Albendazole combined with dexamethasone alleviated inflammation of retina absolutely, but could not recover the damage to the retina and optic nerve, possibly because of the anti-inflammatory effect of dexamethasone which is not potent enough to protect ganglion cells and optic nerve. In contrast, the treatment with TSII-A or CPT combined with albendazole significantly improved ganglion cells damage and optic nerve demyelination. The inflammation of the retina did not completely recover after treatment with TSII-A or CPT combined with albendazole, but the structure of ganglion cells recovered well, and the number of axons in the optic nerve were back to normal, especially in the TSII-A treated group, indicating that albendazole combined with TSII-A is a more efficacious therapy to protect and rescue the damage of the retina and optic nerve in optic neuritis caused by *A. cantonensis*. Up to now, it has been the first time that TSII-A has been reported for the treatment of optic nerve demyelination.

## Conclusion

In conclusion, the present study suggests that TSII-A might be applied as a neurotrophic drug combined with albendazole to treat optic neuritis caused by *A. cantonensis*, and this method is far more efficient than the conventional albendazole - dexamethasone compound, which should be taken into consideration as a highly potential alternative therapy in treating this parasite-induced optic neuritis.

## Abbreviations

AB: albendazole
ANOVA: analysis of variance
CPT: cryptotanshinone
De: dexamethasone
dpi: days post infection
RGC: retina ganglion cells
TSII-A: Tanshinone II-A
VEP: visual evoked potential
